# Service-Learning Programs and Projects for Medical Students Engaged With the Community

**DOI:** 10.7759/cureus.26279

**Published:** 2022-06-24

**Authors:** Jo Ann M Bamdas, Peter Averkiou, Mario Jacomino

**Affiliations:** 1 Medicine, Florida Atlantic University Charles E. Schmidt College of Medicine, Boca Raton, USA; 2 Women's and Children's Health, Florida Atlantic University Charles E. Schmidt College of Medicine, Boca Raton, USA

**Keywords:** adult learning, adult learning theories, medical school curriculum, reflection, medical students, community engagement, service-learning

## Abstract

Introduction

The medical school curriculum has changed from using the term “pedagogy” to framing adult learning theories with the goal of applying knowledge to a clinical situation or real-life experiences. Service-learning programs (SLPs) in medical schools illustrate one of several adult learning principles and practices now used in today’s curriculum that better prepare medical students for working with a variety of patients.

Objective

The researchers’ aim was to assess medical students’ learning experiences while participating with nonprofit organizations during a curricula-designed SLP.

Method

The authors analyzed 60 reflective essays over a three-year academic period from 192 medical students placed in teams of two to four. A qualitative study with a thematic analysis research design was employed in our study. This iterative approach allowed the researchers to identify themes and interpret meaning. The study was completed in 2020 using data from 2017-2020.

Results

Four major themes and one overarching theme emerged that reflect adult learning theories including: (1) transfer learning of one’s skills and knowledge to community and practice; (2) articulate a variety of ways to communicate with multiple, diverse community audiences; (3) employ a creative process for quality improvement strategies; (4) create positive trusting and rewarding relationships that highlight an enhanced level of conduct and professionalism. An overarching theme found was: collaboration emerges almost without forethought. Medical educators may find that replicating this SLP into the curriculum infrastructure provides agency and student buy-in. We established an SLP as part of the medical school curriculum that brings privilege and reward to students and to the community. Reflection provides for meaningfulness from SLP and helps students identify how experiential learning affects their professional development as members of the community and future health care providers.

Conclusion

Implementing SLPs into any medical school curriculum strengthens the adult learning theoretical delivery approach. Disseminating projects and lessons learned to and from the community also showcases experiential learning opportunities for medical students and other professionals. Many aspects of awareness from the medical students’ engagement during the SLP emerged. They learned about specific aspects of community engagement and found it a privilege to give and take many lessons from the experiences and opportunities.

## Introduction

Today, many medical schools in the United States employ adult learning theories to train their students. The goal of adult learning theory is to develop skills for self-directed lifelong learning [1]. The basic concept is that adults learn best when they know why they need to learn something, they can use self-directed learning, the learning involves real-life situations, and the stimulus for learning is internal rather than external [1]. The main objective of medical student learning involves applying knowledge to a clinical situation, especially in a group setting, rather than just hearing something in a lecture format within a classroom. Group learning in an adult experiential learning format also provides opportunities for students to lead activities. Faculty serve as facilitators, providing insights. Learning objectives are established so that each student learns and teaches others. To be able to teach other students, the material must be known well and as a result, education stays absorbed. These learning experiences impact the student to the point that the student can transfer the learning into their future careers.

Service-learning (SL), a form of education promoting social responsibility and service to the community in medical education, fills the research literature, including in seminal journals. While the SL literature holds numerous definitions, our SL program (SLP) has used the Health Professions Schools in Service to the Nation (HPSISN) program [2]. According to Seifer [2], research on medical students engaged in SL provides opportunities to serve the community. These activities identify concerns from which students can learn connections and responses to service and academic coursework as well as a role of themselves as citizens. The key components of SL are reciprocity and reflection [3]. An assurance exists in that these experiences are mutually beneficial to the giver and to the receiver. Researchers of SL also find that these components in higher education have a positive effect on students’ assessments and significantly improve when the program or course ends [4]. In summary, SL stands as a unique experiential approach that trains medical students for providing patient care. In fact, medical schools in the US are required to provide support for SL and community projects, but few medical schools offer structured service-learning [5, 6].

Types of SLPs and courses have been identified in medical schools. In a systematic review of SL from 1998 through 2012, themes and subthemes emerged around program design, implementation, participation, assessment, and student outcomes [7]. Another review of journals noted that only twelve articles or 24.5% of those reviewed were set in the medical field [8]. In a more recent review of the literature, various SL activities available within different disciplines are professional development and practice [9], physical activity programs [10], and student-led free and other types of clinics [11]. 

When students have authentic self-directed interprofessional educational experiences like SLPs and projects, multiple benefits emerge for students [7]. One study showed students describing experiences as motivating and career reinforcing, and deepening commitment to serving future patients with compassion, and fostering the development of professional identities [12]. An additional study found that physicians who participated in medical school service-learning activities indicated that the experiences influenced their professional development and approach to practice [9]. One of the outcomes of an examination of the literature points to a goal being for faculty and students to link service to course objectives [7], which must have clearly stated goals such as “direct, indirect, advocacy, and philanthropic”. Also, a “value-added element to a research project is the opportunity for students to present their findings to the community” [7] with presentations showing the impact of SL along with recommendations for any type of action. While SL curricula in medical education provide myriad benefits to students, faculty, and community members [13], a paucity of literature discusses project development, final assessment, and dissemination processes. Therefore, the purpose of our research study is to discuss the development of our SLP, to evaluate its impact on students, and to disseminate the information to support existing and future health professionals, patients, and families in working together through SLP.

## Materials and methods

**Figure 1:**
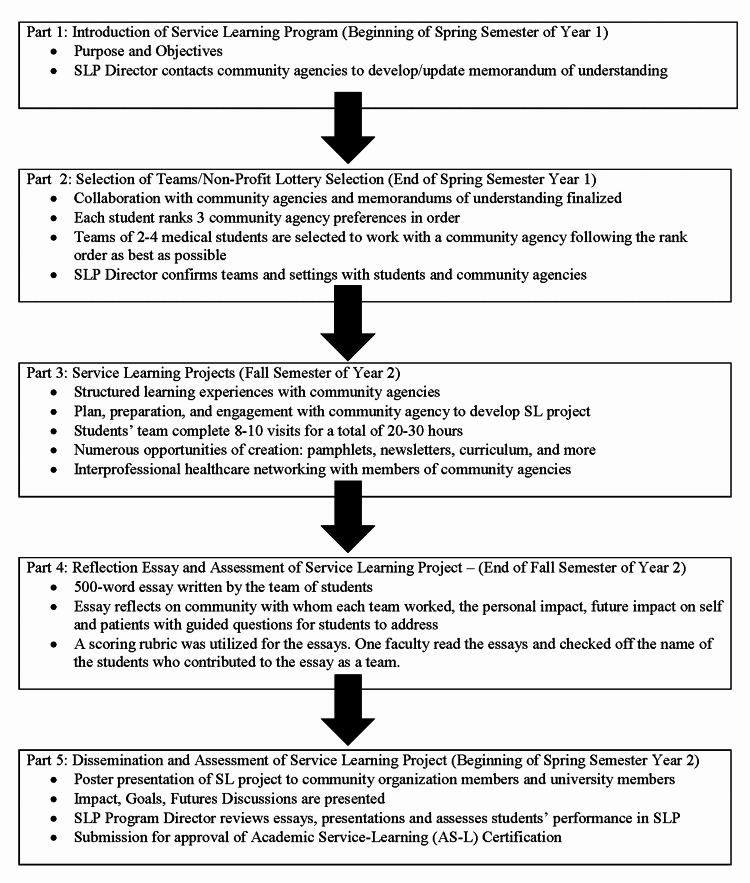
University Service-Learning Project Timeline SLP: Service-Learning Program

Our College of Medicine created an SLP for pre-clerkship students as a component of a course. We created the program for students to work hands-on with a variety of professionals in community non-profit organizations. The aim of the program included exploring skills students employ such as observation, critical thinking, and relevance to community engagement. Our medical schools’ SLP consists of five parts with the students completing an SL Project in Part 3 (Figure 1). 

As part of the assessment of our SLP, students are tasked with completing a five hundred-word narrative essay of their SL project instead of taking a multiple-choice question test designed by faculty. This is yet another adult learning method that moves away from pedagogy. These narrative essays serve as a valuable assessment tool since evaluation drives reflection and meaning.

Population sample and setting

Every year, the entering class of medical students is required to participate in the SLP as part of the medical school curriculum from the spring of Year 1 to the spring of Year 2. The actual number of medical students that participated over a three-year period was 192. Each year, the students were divided into teams that consisted of two to four students. During each academic year of the SLP, our medical students were placed within approximately twenty-three non-profit organizations in many diverse settings. The non-profit organizations were chosen based on community needs; organizations that would provide impactful learning experiences for the medical students and community members served. The nonprofits’ members consist of young children, middle school, and high school students along with parents many of whom have not attended college. During these experiences, our students also collaborated with many professionals including nurses, social workers, psychologists, teachers, pharmacists, physical and occupational therapists, non-profit organization directors, and office managers that work or volunteer their time at the non-profit organizations.

Data collection

The University’s Institutional Review Board granted approval of this qualitative research study. A qualitative approach was chosen in this study because this method allows deep analysis of a text. Qualitative research is a systematic inquiry about educational practices and provides for rich meaningfulness to arise from collected data [14]. Over three academic years (2017-18, 2018-19, 2019-20), we collected 60 medical student teams’ reflective essays. The essays focused on the students’ ability to reflect on their experiences at the end of the project while using the essay as an andragogical tool fostering the students’ reflection capacity. The students had to address the following questions in their essays: (1) the organization and its role in the community; (2) the SL project implemented and what other professionals did they interacted with; (3) how the project has helped the community and the population the organization serves; (4) how this experience has impacted you, and how it will impact your care of future patients and/or the community you will serve; (5) what do you want other students to learn from your experience that will help them in their future. The students in each team participated in writing, reading, editing, and checking for the accuracy of the information in the essay. A scoring rubric was utilized for the essays. One faculty read the essays and checked off the name of the students who contributed to the essay as a team. If all five questions asked of the students were addressed, they received a score of “Satisfactory”. If one or two questions were not addressed, they received a score of “Needs Improvement”. If three or more questions were not addressed, they received a score of “Unsatisfactory”. 

Data analysis and trustworthiness

The essays were de-identified and placed in NVivo 11 software (QRS International, Ruggell, Liechtenstein) and coded independently by the first author. None of the 60 essays were excluded from our analysis. Next, each author independently coded one academic year’s verbatim essays. Each researcher then reviewed all codes from all three years. It has been indicated that decisions about the approach to coding are critical, yet these are seldom discussed in the literature [15]. Once the coding process ended, the authors met to discuss the code words and phrases that emerged. The team members adopted a quasi-version of Olson et al. [16] constant comparative method due to the fact “that multiple researchers were performing data analysis and meaning-making”. Upon completion of the coding, we performed a thematic analysis. This process typically involves a deeper level of interpretation of the data that is both deductive and inductive [17]. A consensus process was used throughout to crystallize theme statements. Finally, we identified key statements that aligned with the students’ reflections. Each author was invested in and owned each responsibility within the program and the analytical process [18]. Through this process and the three years period under study, we were confident as researchers that we uncovered the trustworthiness of the research study results with credibility, transferability, dependability, and confirmability [19].

## Results

Four major themes and one overarching theme emerged. The findings illuminate the students’ development during this SL program. 

Theme 1 - Transfer learning of one’s skills and knowledge to community and practice

Most medical students’ essays indicated that they performed multiple roles including educator, teacher, tutor, and/or mentor. Students explained that using the materials learned in their medical school curriculum had helped them to transfer their own knowledge, skills, and abilities to an organization’s members. Some of the vital skills mentioned that suited their needs included interpersonal and communication skills, problem-solving, clinical reasoning, logistical planning, and role modeling. Medical students’ abilities to take on the responsibility of role modeling were a dominant take-away from their experiences throughout their SLP. They appreciated the importance of carrying on the lessons learned from their experiences into the roles each would have as future physicians. Many medical teams expressed similar sentiments to the following: "(This organization) has given (us) the platform to reach back and pull the next generation of minority healthcare professionals forward. Together, we can continue to encourage, instruct, and motivate students from all backgrounds to successfully pursue a rewarding life in the field of medicine! It is our responsibility to carry the lessons learned from these types of experiences out into the real world as smart, humble, and most of all, caring physicians."

Theme 2 - Articulate a variety of ways to communicate with multiple, diverse community audiences

Ways of communicating emerged involving the articulation of active interaction to various audiences by relaying healthcare information in engaging, innovative ways. Most described the active process of learning and teaching to their audiences. Student teams found innovative ways to engage with the individuals at each organization. Each student took complex terminology, reduced it into manageable, digestible facts, and recommended resources. Students discovered simple ways to meet the eyes of their audience, even as they crouched down to the person with whom they were communicating. Students critically thought about their audiences, and how to best approach them to gain maximum communication. Listening, asking questions, and sustaining conversations took on a new life, as they were introduced to the audiences each served. This team reflected on the joy of communicating through the spirit of dance: "The clients especially loved dancing, so we would dance with them every week to the point where we had a few songs that were almost choreographed. Our hope is that when the clients hear those songs in the future, they dance again, remembering our talks about the importance of exercise, improved health."

Theme 3 - Employ a creative process for quality improvement strategies

Program creativity strategies occurred, which made for more effective team-based leadership practice and services to diverse community members. The idea of creativeness placed within the teams is at the core of SLP. Each team focused on brainstorming, decision-making, planning for a creative process, and improving the process of building services and resources for each diverse community. Each student and team exhibited high levels of educational leadership too. Undergirding the student teams’ plans for their goals to complete projects stayed the concept of instilling in the organization’s members skills and abilities to see oneself as doing or being whatever one wants. Students practiced showing the audience building confidence: “If you want to be a doctor, or whatever role you want in life, you can achieve it. If you want to be a leader, then you can be a leader”.

Theme 4 - Create positive trusting and rewarding relationships that highlight an enhanced level of conduct and professionalism

This final and perhaps more important theme centered on the values and ethics of creating positive, trusting, and rewarding relationships that highlight an enhanced level of ethical conduct and professionalism. Students said they would never forget the building of relationships with their community. The impact came from the member’s diverse backgrounds and individual differences of populations that they came to embrace. Students came away at the end of their projects with a level of respect, inclusion, and professionalism that would affect them personally and professionally. The following quote sums up most of the reflective expressions: “Serving the community is about being open with each other as equals and refusing to forget that we belong to each other as members of the same community. We each have our own gifts and talents to share. If we invest the time and energy into developing relationships with the people around us, the reward is great.”

Overarching theme - Collaboration emerges almost without forethought

We identified an overarching theme. The SLP brought reward from the privilege of working with diverse community organizations and their members. The students’ experiences showed collaboration with community members, the organization’s leadership, the team members, and indeed the medical school’s students and faculty. This engagement in SLP related to positive, rewarding relationship building, employing and creating leadership and service, and listening to what the population desires for themselves. This transferred into medical knowledge, skill, and attitude as noted in the following reflection: "During one of the early sessions, we noticed that the children were being unruly. At first, we assumed this to be a sign of disrespect, and only tried to speak louder and to impose discipline. However, at the conclusion of the session, one of our team members thought to ask if any of the children wanted to go outside next session. Immediately, every hand in the room shot up, and every eye lit up with an energy we had not seen all day. When planning the next session, we threw out all our preformed . . . planning sessions . . . from that day on, we allowed students to learn . . . with fun."

## Discussion

We began this study desiring to know the value and benefits of our SLP to the medical students and the community’s nonprofit organizations. We also wanted to know how we could observe and measure their learning as adults. We discovered that the students appeared to greatly value-creating their own SL project through their learning experiences in the community. The students valued their individual and teams’ ability through the SL project to plan, construct, teach, mentor, assess themselves, and to use what they learned in their medical school curriculum. These abilities are clearer to the researchers through multiple reviews of each year’s student teams’ reflections. According to Cunningham et al. [20], narrative reflection in medical education provides an opportunity for self-awareness and empathy, along with the construction of a holistic professional identity.

“Recent literature reviews on learning transfer indicate that a large percentage of adult learning does not successfully transfer” out [21]. But our medical students successfully produced reflections stating that they would “transfer learning” from medical school including newly learned knowledge, skills, and behaviors into a lived context as described by Burke & Hutchins [22]. A major theme indicated that students would transfer medical SLP experiences when engaged with diverse community member organizations, their administrators and staff, their members, and their families through activities.

The wearing of the white coat often becomes a “transformational or transformative learning experience” for developing medical students into future physicians. This is an experience that is triggered by something else or focuses on an idea that learners can adjust thinking based on new information [23] and “real life” experiences. Working with communities within an SLP, medical students and community members do experience moments of such transformational learning. The nonprofits’ members consist of young children, middle school, and high school students along with their parents many of whom have not attended college. The community SLPs open the possibility for underserved/at-risk children to think about becoming a physician or other healthcare professional, where previously the thought may never have been conceivable.

Students should reflect on how adult learning theories specifically experiential learning, self-directed learning, and meaning-making like transformative learning affects their professional development as a member of global, national, and local communities. Our students enjoyed collaborating with the community’s staff and leaders at the different organizations they served. Students clearly benefit from understanding themselves, and more importantly, from their interaction with the community [20]. Specifically focusing on actual experiences with families and individuals in communities makes a difference [24]. Research has shown that student engagement with the community and SLP, or any other type of community-based participation, provides a space for academic knowledge and skill and challenges to be applied and tested during learning [25]. Over the three years studied, this cohort of medical students found it a privilege to give and take many lessons from the experiences and opportunities in the medical school’s SL program. Many students described these experiences as “life-changing”, commenting that “the experience influenced their choice of specialty”.

Several limitations exist in this study. The findings cannot be generalized over all medical students’ SL experiences with all community nonprofit organizations. This study is also limited to the reflective essay as a method of assessment. There are several interprofessional education and collaborative practice and service-learning instruments that could be used to measure quantitative data in the future.

A final benefit of our program and to others seeking to grow SLPs is in providing volunteer hours that become recorded onto students’ transcripts. We encourage our medical students to add the SLP to their curriculum vitae, which makes them better qualified applicants, especially for those students pursuing post-medical school training in community service-oriented specialties. 

## Conclusions

Implementing SLP into any medical school curriculum strengthens the adult learning theoretical delivery approach. The collaborative moments with community organizations’ leaders and families during the team projects resulted in two major areas of transformational learning: the importance of the educational materials using a play or entertaining lesson approach, and the value of learning the cultural aspects or the diversity of the community’s members and their families. The students learned about specific aspects of community engagement. They found it a privilege to give and take many lessons from the experiences and opportunities they had. Medical students trained with adult learning theories and SLP ensure that the world will be full of physicians who will fill multiple roles and responsibilities rather than as a one-dimensional healthcare provider.
